# Bioinformatics analyses of retinoblastoma reveal the retinoblastoma progression subtypes

**DOI:** 10.7717/peerj.8873

**Published:** 2020-05-21

**Authors:** Manjing Cao, Sha Wang, Jing Zou, Wanpeng Wang

**Affiliations:** 1 Eye Center of Xiangya Hospital, Central South University, Changsha, Hunan, China; 2 Hunan Key Laboratory of Ophthalmology, Changsha, Hunan, China; 3 Department of Ophthalmology, The Second Xiangya Hospital of Central South University, Changsha, Hunan, China

**Keywords:** Retinoblastoma, Progression, Subtypes, Clustering, LRRC39

## Abstract

**Introduction:**

Retinoblastoma (RB) is one common pediatric malignant tumor with dismal outcomes. Heterogeneity of RB and subtypes of RB were identified but the association between the subtypes of RB and RB progression have not been fully investigated.

**Methods:**

Four public datasets were downloaded from Gene expression omnibus and normalization was performed to remove batch effect. Two public datasets were explored to obtain the RB progression gene signatures by differentially expression analysis while another two datasets were iterated for RB subtypes identification using consensus clustering. After the RB progressive subtype gene signatures were identified, we tested the diagnostic capacity of these gene signatures by receiver operation curve.

**Results:**

Three hundreds and forty six genes that were enriched in cell cycle were identified as the progression signature in RB from two independent datasets. Four subtypes of RB were stratified by consensus clustering. A total of 21 genes from RB progression signature were differentially expressed between RB subtypes. One subtype with low expression cell division genes have less progression of all four subtypes. A panel of five RB subtype genes (CLUL1, CNGB1, ROM1, LRRC39 and RDH12) predict progression of RB.

**Conclusion:**

Retinoblastoma is a highly heterogeneous tumor and the level of cell cycle related gene expression is associated with RB progression. A subpopulation of RB with high expression of visual perception has less progressive features. LRRC39 is potentially the RB progression subtype biomarker.

## Introduction

Retinoblastoma (RB) is a childhood cancer of the retina that is usually caused by inactivation of the biallelic gene of the RB1 tumor suppressor gene (Soliman et al., 2017). A total of 40% of patients with RB have genetic susceptibility due to germline mutations in RB1 (Kamihara et al., 2017). Hereditary patients need only inactivate RB1 in vivo to develop RB so they are usually affected bilaterally.

Understanding the tumorigenesis of RB cells is critical to understand the progression of RB so that personalized care could be applied for individuals. Alterations in the RB pathway due to mutations in the RB1 gene mutation or other related signaling pathway components (e.g., D-type cyclin, CDK4 or p16INK4A) are common in RB (Bartkova et al., 1996). Although these mutations are primarily thought to affect cell cycle regulation, RB1 has also many other regulatory factors, including regulation of chromosomal stability, senescence, and cell differentiation (Indovina et al., 2013). Abnormal regulation of cell cycle arrest and loss of differentiation is likely relevant in the context of RB development and particularly in RB1 mutation-related RBs. Understanding the molecular expression of RB at different levels of malignancy will enable patients to benefit from the targeted therapeutics. Previous studies have shown that inhibition of EGFR pathway in RB can effectively reduce the progression of RB, highlighting the clinical utility for RB (Witkiewicz & Knudsen, 2014). Understanding the molecular pathogenesis of RB can also elucidate other RB1 mutated tumors. These tumor types also show high frequency of RB1 gene mutations/deletions (instead of cyclin/CDK/CDKI mutations), such as small-cell lung cancer, bladder cancer, breast cancer and hepatocellular carcinoma (Ahn et al., 2014; Rubio et al., 2019; Sutherland et al., 2011; Witkiewicz & Knudsen, 2014).

Although the non-genetic form of RB is usually caused by somatic cell inactivation of two RB1 alleles, a subtype of RB has recently been described that lacks the RB1 mutation but shows a high level of amplification of the oncogene MYCN (Rushlow et al., 2013). In addition to the initial hit (RB1 mutation or MYCN amplification), RB may require additional DNA mutations to tumorigenesis and development (Dimaras et al., 2008). Common chromosomal changes observed in RB are the increase in chromosomal regions 1q, 2p and 6p, and loss at chromosome 16q (Theriault et al., 2014). In addition to copy number analysis, several gene expression studies have been published (Chakraborty et al., 2007; Ganguly & Shields, 2010; Kapatai et al., 2013; McEvoy et al., 2011). It has been suggested that RB has a similar expression profile and expresses genes involved in various differentiation programs (McEvoy et al., 2011). However, in another recent study (Kapatai et al., 2013), RB is likely a heterogenous disease where two different RB subtypes were identified based on gene expression profiling. One group expressed genes associated with a range of different retinal cell types, indicating progenitor cells of origin, while the other group exhibited high expression of conical photoreceptor related genes, suggesting derivation from cone photoreceptor cell precursors (Xu et al., 2014). Therefore, based on the RB molecular expression profile, subtype analysis of RB provides a further understand the biological development mechanism of RB, as well as the optimal treatment of children and families.

Stratification of RB revealed the heterogeneity in RB and the potentially optimal therapeutics for patients with different molecular profiles. However, whether one of subtypes of RB is related with RB progression has not been fully elucidated. Here, we use bioinformatics analysis of public datasets to identify genetic features that are critical in the progression of RB. We found four subtypes in RB according to their transcriptomics. The expression of RB progression genes in these four RB subtypes was inconsistent, suggesting a different treatment approach for RB subtypes. Subtype4 of RB that featured by five signature genes had less malignant RB gene expression.

## Materials and Methods

### Public datasets

Two public datasets (GSE97508 and GSE110811) investigating the gene expression of normal retina and RB were queried and the normalized gene expression data was downloaded from Gene expression omnibus (GEO) for RB progression signature iteration using “GEOquery” package. A total of 131 samples were acquired from GEO and the sample information were also downloaded. Another two independent public dataset (GSE29683 and GSE59983) were obtained from GEO for subtype stratification of RB. All probes were converted to official gene symbol names for downstream analysis. For each official symbol gene targeted by multiple probes, the median expression of all probes regarding to the gene was used for further analysis.

### Data normalization and exploration

For generating a comprehensive dataset, preprocess by normalization was utilized to remove the batch effects. Boxplots of all genes of each sample were utilized for visualization of removal of batch effects. Principal component analysis (PCA) was employed for dimension reduction exploration. The distances of samples were determined by the root-mean-square deviation (Euclidean distance) for the top 2,000 genes. Heatmaps were used for visualization of the indicated genes expression level per sample.

### Differentially expressed genes

Considering the distinct platforms of our included datasets (GSE97508 and GSE110811), we performed the Differentially expressed (DE) genes analysis separately. To increase the validity of RB progression signatures we proposed here, we shortlisted a common gene list by overlapping the DE genes generated from two datasets. Agglomerative hierarchical (Ward, complete-linkage, average-linkage and McQuitty) clustering was performed on inverse absolute Pearson correlations (Verhaak et al., 2010; Zhou, Xu & Liu, 2017). Differential expression testing was performed on linear modeling of indicated (co-)variates on expression values by limma Bioconductor package (Ritchie et al., 2015). *P*-values generated from limma modeling were corrected for multiple hypothesis testing by Benjamini & Hochberg false discovery rate (FDR) adjustments. The FDR-adjusted *P*-values < 0.05 were considered significant.

### Subtypes identification

Hierarchical clustering with agglomerative average linkage was performed in this study, as our basis for consensus clustering, to detect robust clusters. The distance metric was 1-(Pearson’s correlation coefficient) was utilized for variances detection between samples. SigClust was performed to establish the significance of the clusters in a pairwise fashion. All subtypes identification was performed using “ConsensusClusterPlus” Bioconductor package. The overlap of genes was plotted by Venny (https://bioinfogp.cnb.csic.es/tools/venny/).

### Gene annotation analysis

The identified DE genes were annotated by Gene Ontology and Kyoto encyclopedia of genes and genomes (KEGG) analysis using “clusterProfiler” Bioconductor package when the candidate genes were more than 10 (Yu et al., 2012). If the candidate genes were less than 10, the pathways of the indicated genes were identified by Metascape. FDR < 0.05 was considered as statistical significance.

### Diagnostic biomarker capacity test of RB progressive gene signatures

We applied receiver operating characteristic (ROC) curves methods to identify the discrimination capacity of RB progressive gene signatures. Area under the curve (AUC) was employed as the accuracy of the indicated marker.

### qRT-PCR

We conducted a qRT-PCR for the five RB progressive gene signatures in 15 RB and 15 invasive RB tissue samples. All RB tissue samples were stored in the liquid nitrogen immediately after removed from patients and washed by PBS once. After extracted from RB tissue samples by TRIZOL, RNA of each sample was quantified by NanoDrop Spectrophotometer ND-1,000. RNA integrity was determined by 2100 Bioanalyzer (Agilent, Santa Clara, CA, USA). Once RNA passed the integrity (OD_260_\OD_280_ > 2.0), 1 µg of RNA was used for complementary DNA reversion. Complementary DNA was synthesized by qScript cDNA Super Mix (Quanta Biosciences, Salt Lake City, UT, USA) under the following cycle parameters: 4 min at 25 °C, 30 min at 42 °C, 5 min at 85 °C, hold at 4 °C. After complimentary conversion, we performed the qRT-PCR in 10 µl reaction system with 1 ng cDNA template and analyzed the PCR results for each sample using the Perfecta SybrGreen (Quanta Biosciences) in Roche LightCycler 480. The PCR efficiency was tested by standard curve. Once the standard curve showed the *R*^2^ is larger than 0.99, we performed PCR accordingly. No template controls were included as the control in each PCR reaction. Melting curves were used for identification of PCR products. The RT-PCR reaction was conducted under the following cycle parameters: 5 min at 95 °C for start, 35 cycles (20 s at 96 °C, 30 s at 55 °C, 1 min at 72 °C) for amplification and at 4 °C for holding. Actin was utilized as the endogenous control. The relative expression of LRRC39 in each sample was presented as fold change (log_2_ 2^−ΔΔCT^) for statistical comparison. *P*-value less than 0.05 was regarded as statistical significance. Primers include: Actin (F: 5′-TATCCCTGTACGCCTCT-3′; R: 5′-AGGTCTTTGCGGATGT-3′), LRRC39 (F: 5′-CAACAAACTTGAACAACTTCCTGA-3′, R: 5′-GCAAGCATGTTATTTCATTTCG-3′)). These primers are verified in previous study (Will et al., 2010).

## Results

### Differentially expressed genes were identified as RB progression signatures

**Figure 1:**
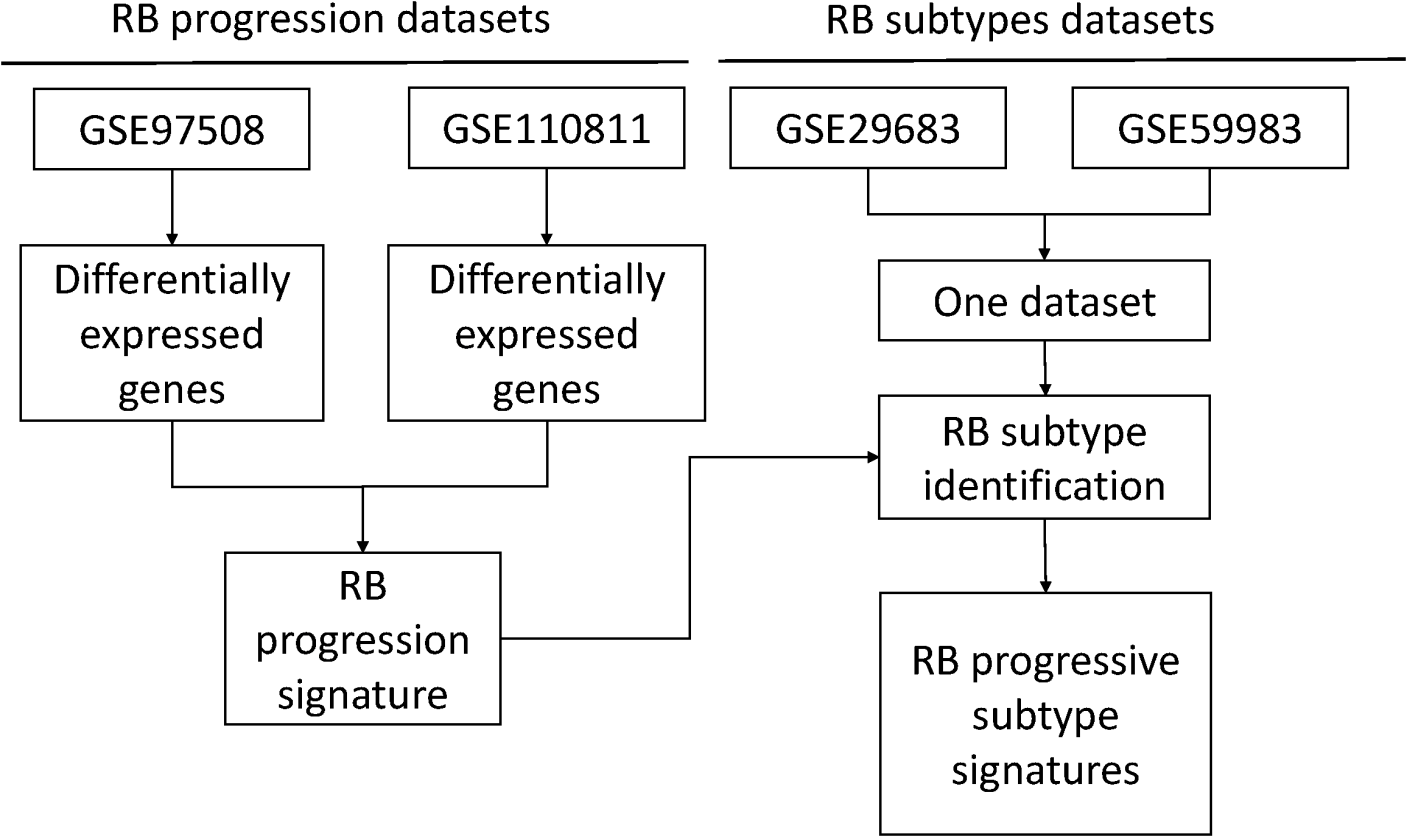
A heatmap of differentially expressed genes in GSE110811.

**Figure 2:**
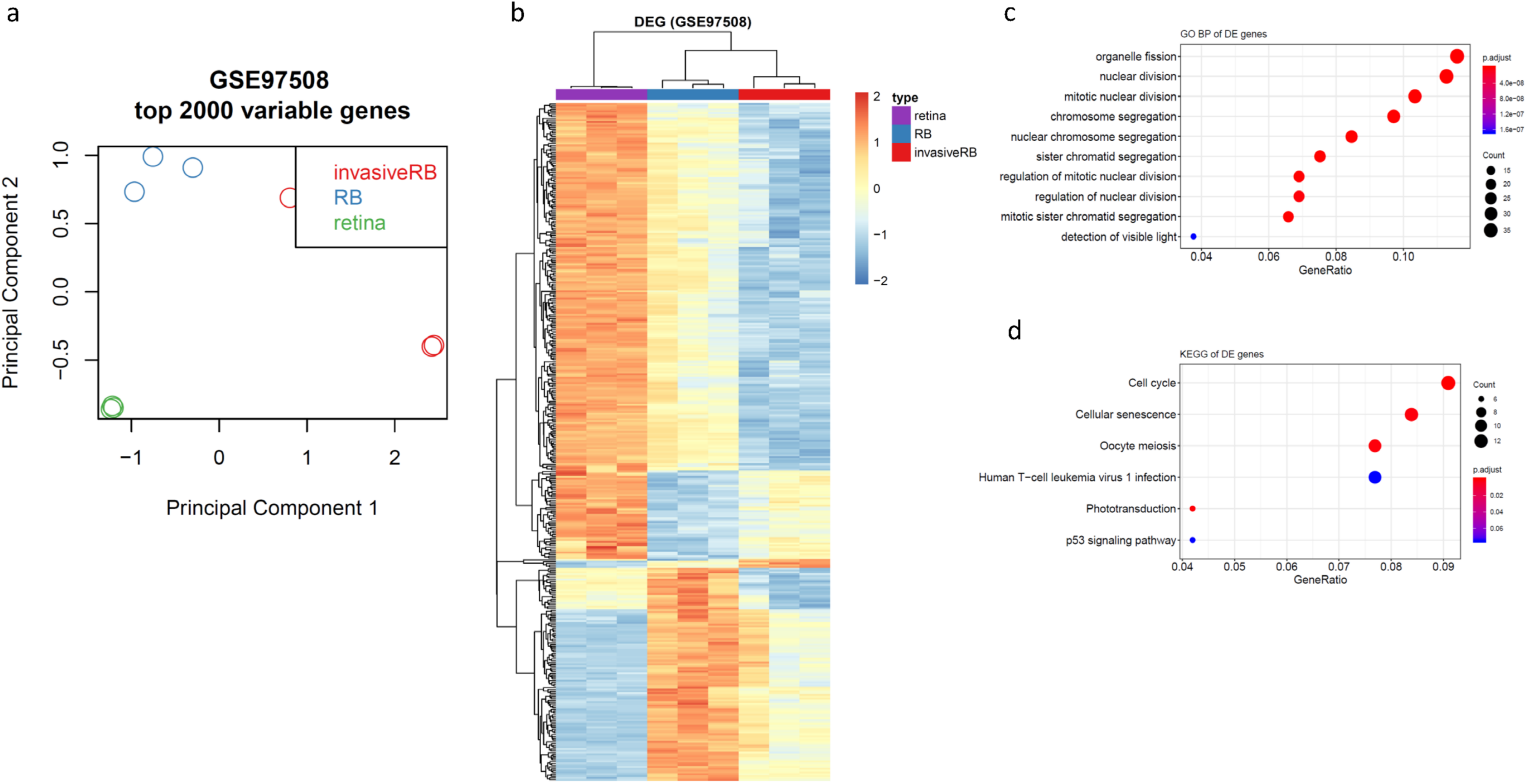
A combination of two independent public datasets for RB subtype identification. (A) Boxplots of the gene expression of each included samples before (left) and after (right) batch correction. Green, GSE29683; blue, GSE59983. (B) PCA plots showing the variance of RB labeled by batches (left) or subtypes (right) using top 2,000 variable genes. Each dot represents each RB samples. (C & D) Bubble plots of GO and KEGG analyses of the differentially expressed genes. Color of bubbles represents the adjusted *p* values while the size indicates the counts of genes are enriched in the given pathway.

To obtain the RB progression signatures, we identified two DE genes lists regarding RB progression from two independent public datasets (Fig. 1). We firstly explored the gene expression using the GSE97508 dataset that containing normal retina, RB and invasive RB to investigate the heterogeneity of RB in different stages in terms of gene expression. PCA plot showed there were large differences between normal retinas, RBs and invasive RBs, suggesting gene signatures could be identified during the progression of RB (Fig. 2A). Then, we performed the differential gene expression analysis for this dataset to shortlist the genes were associated with RB progression. A total of 376 genes was identified as RB progression signatures when the threshold of differential gene was set as log fold change larger than 1 and FDR less than 0.05 (Fig. 2B; Table S1). Gene Ontology analysis identified these RB progression signatures were enriched in chromosomal region, mitotic nuclear division, microtubule binding and microtubule motor activity. Similarly, cell cycle and cell division pathways are enriched in these RB progression signatures from the analysis of KEGG (Fig. 2C).

Similarly, we performed another DE genes analyses on public dataset GSE110811 (Fig. 1) using the identical tools and statistical threshold as we mentioned in the analysis for GSE97508. A total of 123 DE genes were obtained from these dataset (Fig. S1; Table S1). A common gene list was generated by overlapping these two DE genes and described as RB progression signature (Table S1).

### Consensus clustering identifies four subtypes of RB

**Figure 3:**
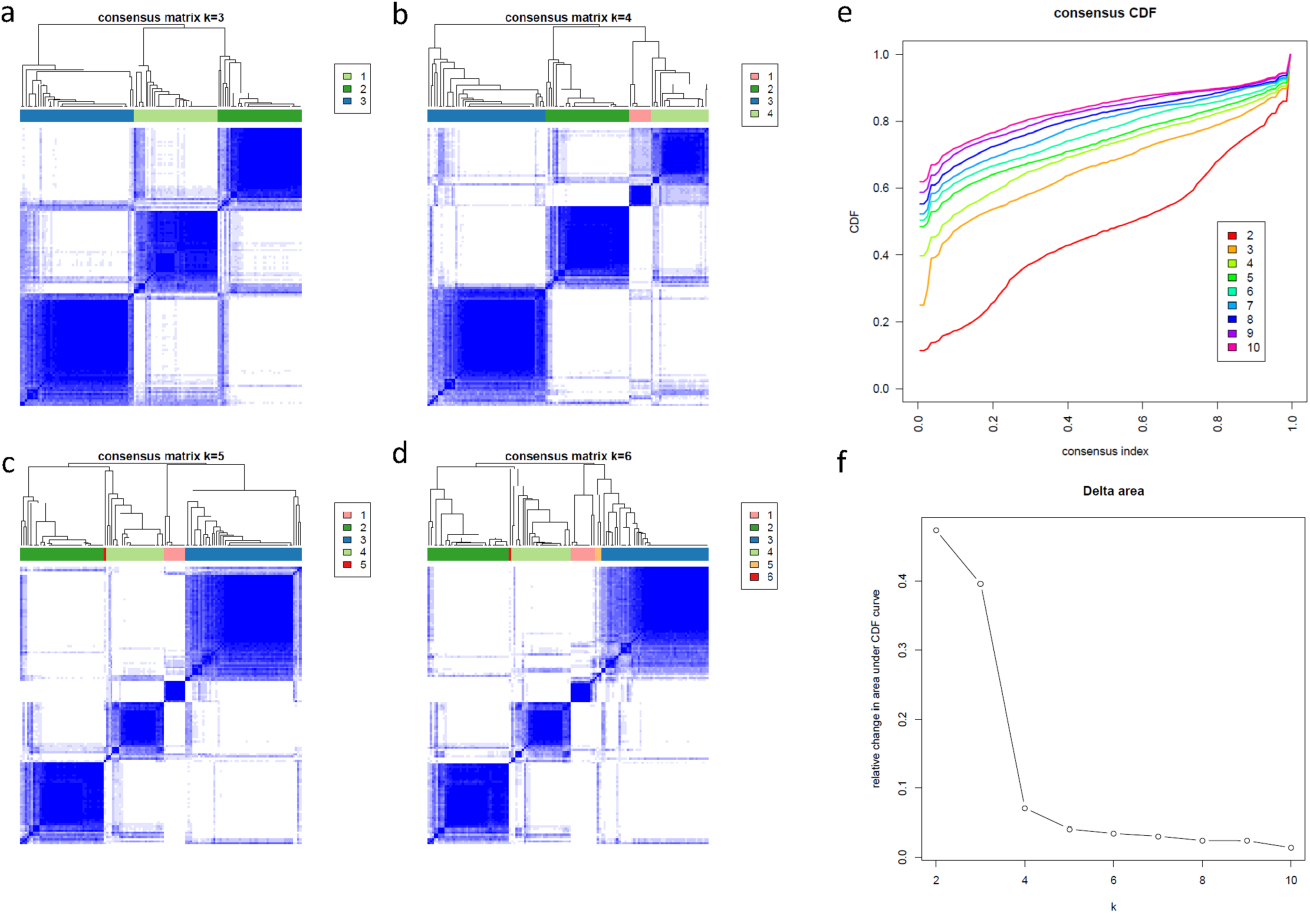
Identification of four subtypes in RB. (A–D) Consensus clustering matrix of 131 RB samples for *k* = 3 to *k* = 6. (E) Consensus clustering CDF for *k* = 2 to *k* = 10. (F) Delta area plot showing the relative change in area under the CDF curve from *k* = 2 to *k* = 10.

As a high heterogeneity of RB was demonstrated by two previous studies, we sought to investigate whether the progression signatures were enriched in specific subgroups of RB, indicating one or specific subgroup RB were prone to be more progressive and more adjuvant therapeutics were applied in diagnosis. To determine the subgroups in RB, we first downloaded and combined two public available datasets (GSE29683 and GSE59983). Because there was a significant batch effect between these two datasets, we performed a batch effect correction before data exploration (Fig. S2A). A total of 131 RB samples were included in one uniformed dataset. Principle Component Analysis revealed there was significant variance between RBs, suggesting stratification for RB is required for further understanding the progression of RB (Fig. S2B). Using this dataset, we filtered all samples into 2,000 genes with high variable expression across the two datasets. Consensus average linkage hierarchical clustering of 131 samples and 2,000 genes identified four robust clusters with clustering stability increasing for *k* = 3 to *k* = 6, but not for *k* > 4 (Figs. 3A and 3B). We also observed a significant decrease of delta area for *k* = 4 subtype clustering and delta area entered into plateau when k was larger than 4, which confirmed stratifying RBs into four subtypes is sufficient for illustrating the variance between RBs (Fig. 3C).

**Figure 4:**
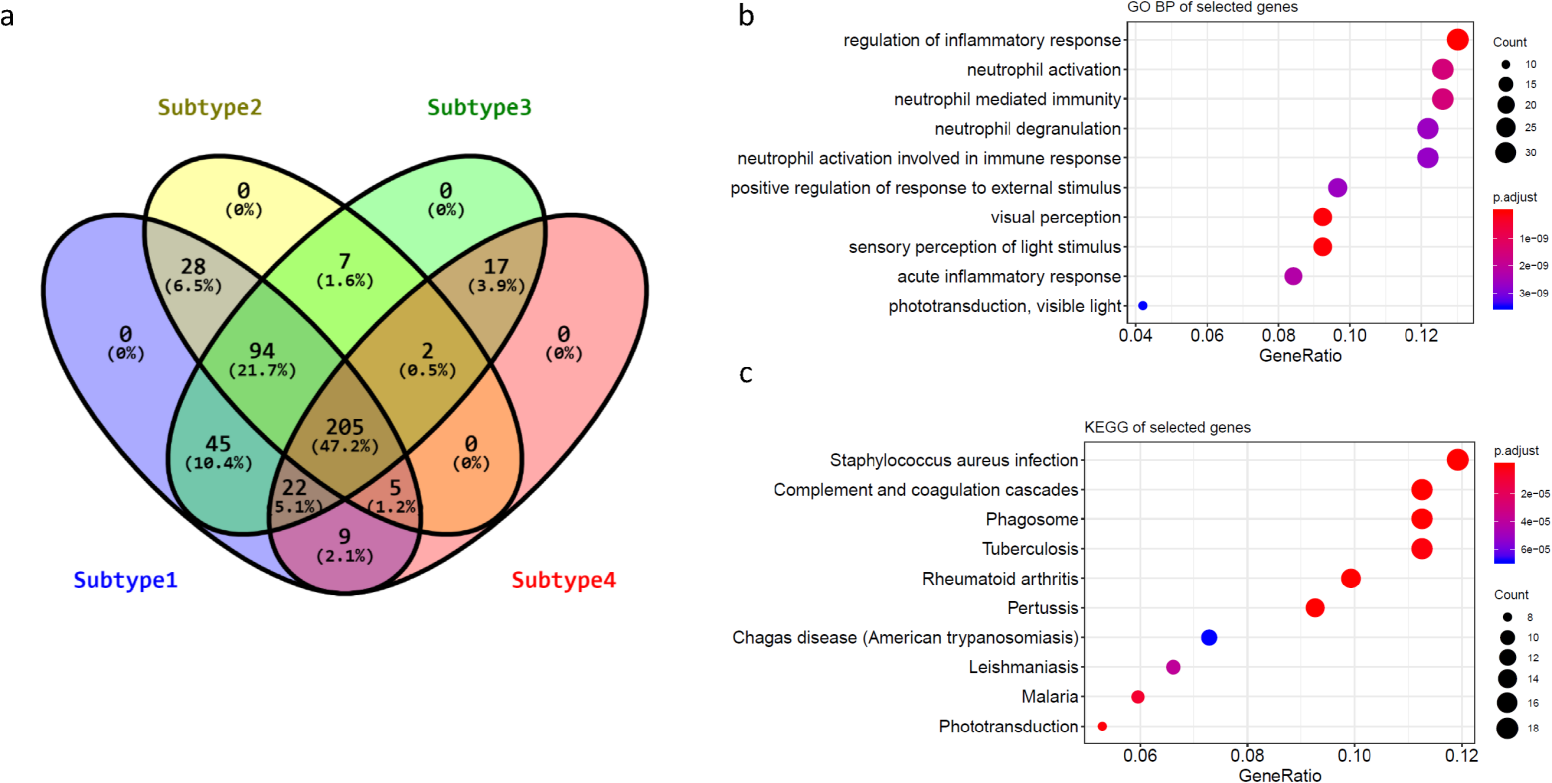
Subtype specific genes and their enriched pathways. (A) The Venn diagram showing the overlap and subtype specific genes in each RB subtype. (B and C) Bubble plots of the enriched GO and KEGG pathways of subtype4 specific genes.

To further understand the biology of these four subtypes of RBs, we determined the subtypes signature core genes and pathways by differentially expression and gene ontology analyses. We utilized “Limma” package to identify the DE genes between the four subtypes of RB. In comparison, each subtype was tested against the other three subtypes separately. The number of DE genes for each subtype was 408 (subtype1), 341 (subtype2), 392 (subytpe3) and 260 (subtype4), respectively (Table S2). A total of 436 genes were identified as DE signatures (Table S2) after removal of the duplicates in comparisons. Venn diagram was used to demonstrate the overlap of DE signatures in each subtype. Surprisingly, nearly half DE genes (205, 47.2%) were presented in all subtypes of RBs and none DE gene was shown as subtype specific (Fig. 4A), suggesting RB subtypes had distinct patterns in term of these shared genes. To further understand the biological functions of these subtype genes, Gene Ontology and KEGG pathways analyses were performed. Due to the large overlap of these subtypes’ genes, most enriched pathways were also shared with groups, suggesting these pathways were the key features between RB subtypes and their expression levels could be potentially the biomarkers for subtype identification in RB (Fig. 4B; Fig. S3). We also validated these subtype specific genes were capable of discriminating invasive RB from normal retina and RB (Fig. S4).

### Twenty-one RB progression signatures were differentially expressed in four RB subtypes

**Figure 5:**
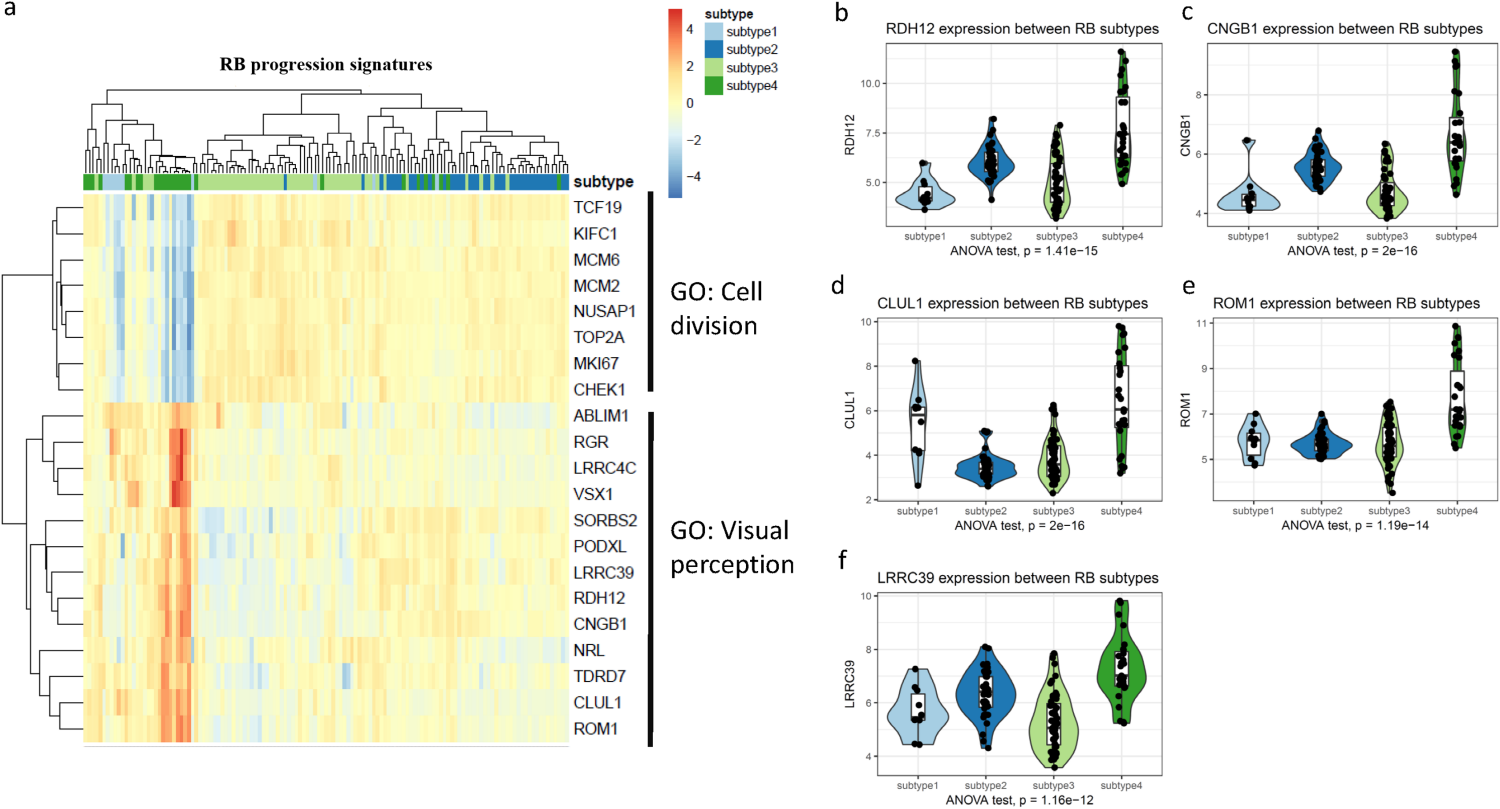
Identification of RB progressive subtype signatures. (A) A heatmap of progression signature genes in RB subtypes. (B–F) Violin plots of the expression of five RB progressive subtype signatures between four RB subtypes. ANOVA was performed to test the differences between groups.

Given the subtype specific genes were identified by differential expression analysis, we next investigated whether these RB subtype specific genes were involved in RB progression. From the RB progression signatures, we identified twenty-one genes were associated with RB progression (Fig. 5A). Clustering of all RBs on these 21 genes revealed three main large subgroups. Interestingly, the subgroups clustered by 21 RB progression signatures was largely overlapped with the subtypes of RB, suggesting distinct malignancy between RB subtypes. Most significantly up/down regulated RB progression signature genes were enriched in subtype4, indicating the most progressive RBs were likely absent in subtype4.

To further investigate these 21 RB progression signatures in RB subtypes, we performed gene annotation analyses for these 21 genes by Metascape (Fig. S5). Gene annotation analyses identified these 21 gene progression signatures were clustered into two main groups: cell division and visual perception. Notably, cell division related genes were extremely low expressed while visual perception related genes were remarkably up-regulated, suggesting subtype4 RB was the less progressive subtype of the four RB subtypes (Fig. 5b; Fig. S6; Table S3).

To shortlist the RB progressive subtype signatures, we overlapped the RB subtype specific genes with RB progression signatures. Five genes remained as RB progressive subtype signatures (CLUL1, CNGB1, ROM1, LRRC39 and RDH12). Notably, these five RB progressive subtype signatures were significantly up-regulated in subtype4 RB and normal retina (Fig. S6), suggesting patients with subtype4 RB likely had a less progression. To cross-validate our five RB progressive subtype signatures, we compared our subtype genes with the previous study that identify three subtypes of RB. All the five genes were also identified as the DE genes, suggesting these five genes were robust for subtype classification and consistent with other studies (Fig. S7; Table S4).

### RB progressive subtype signature was potentially the diagnostic biomarker for RB progression

**Figure 6:**
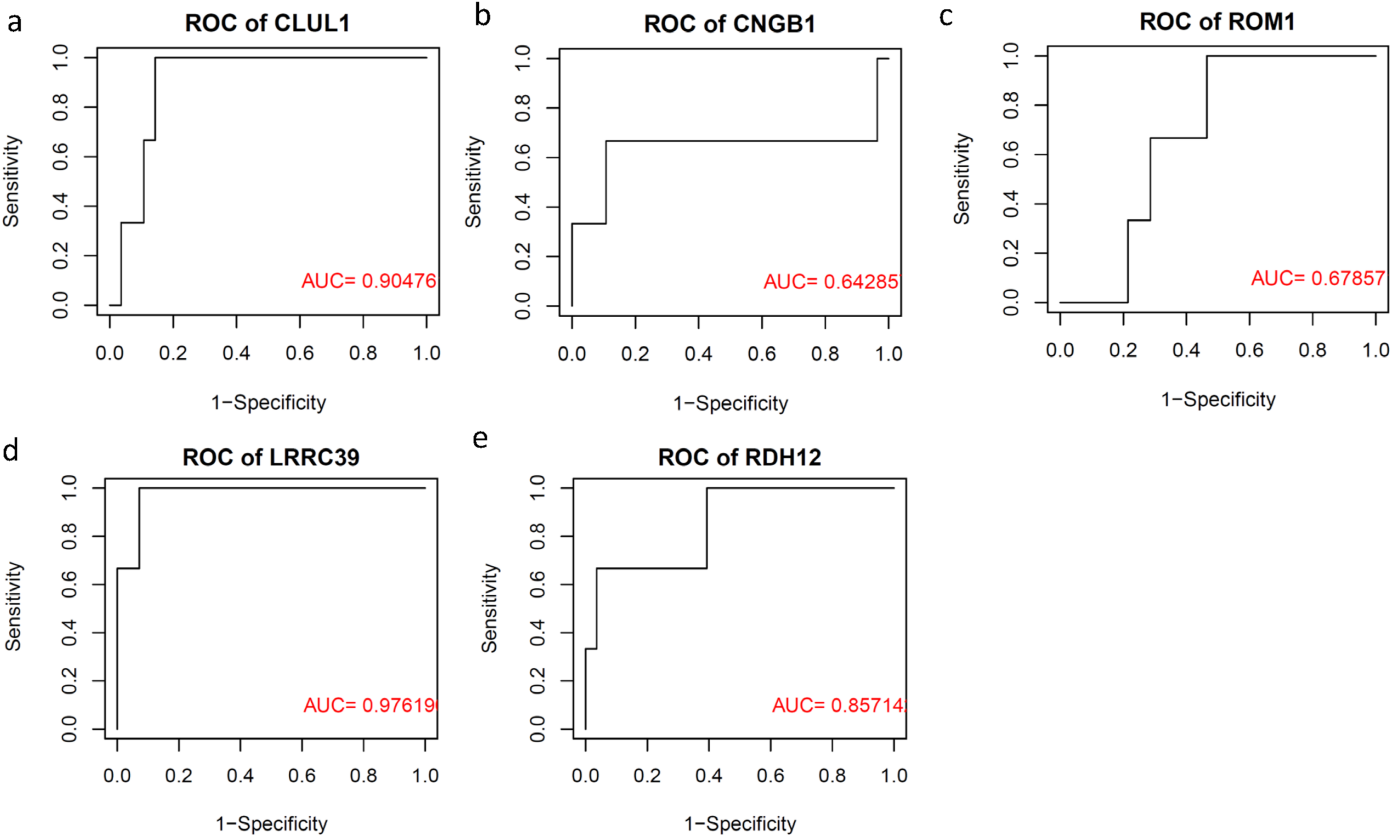
ROC curves (A–E) of five RB progressive subtype signatures showing the discrimination ability of identifying the progression of RB.

To validate the clinical utility of RB progressive subtype signatures, we tested whether these five genes had sufficient capacity of differentiating less progression RBs from all RBs using dataset GSE110811. ROC curves were plotted to demonstrate the diagnostic capacity of each signature gene as a biomarker (Fig. 6). We found LRRC39 had the most outstanding diagnostic capacity of these five genes with 0.98 of AUC, suggesting LRRC39 might be the best candidate for RB progression and subtype stratification biomarker.

**Figure 7:**
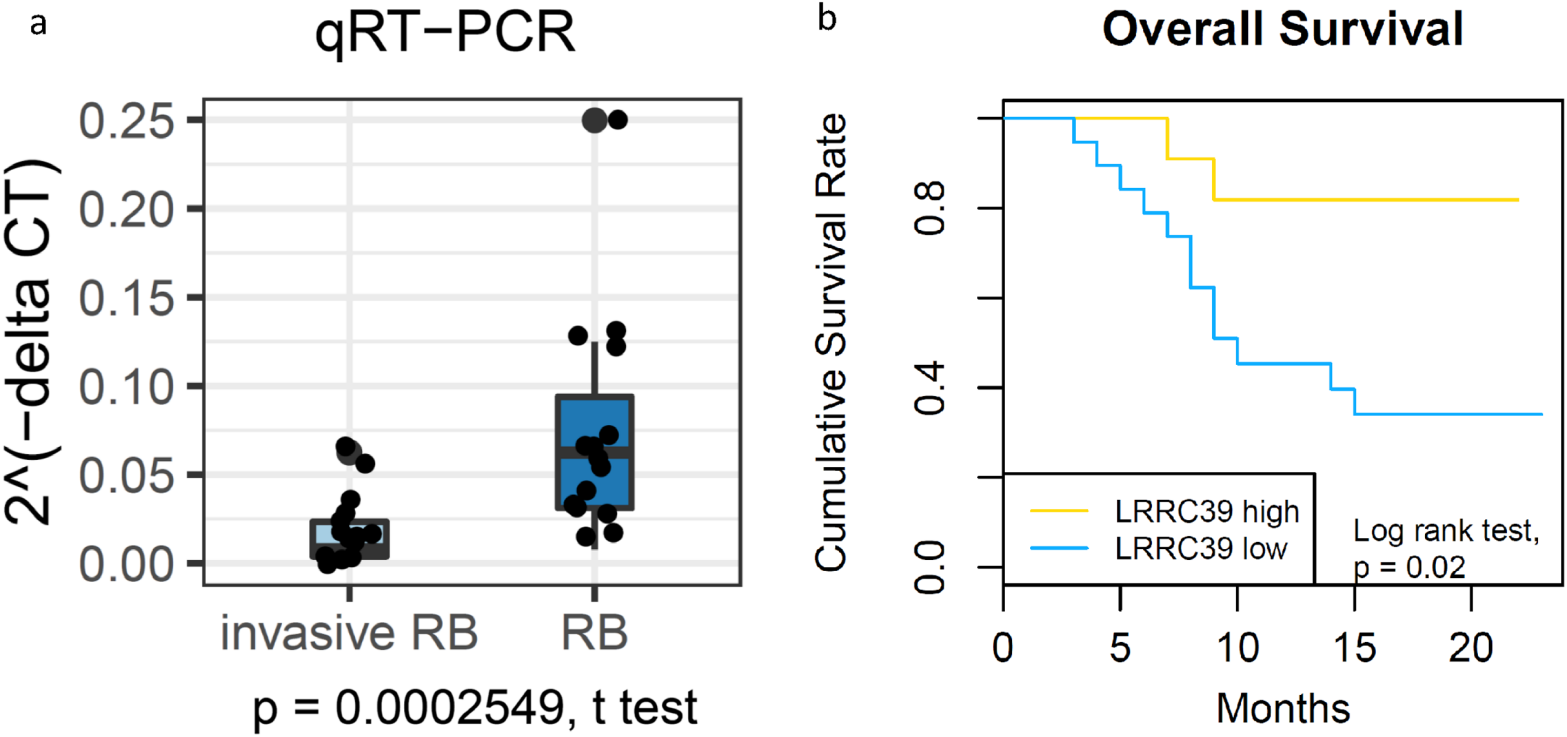
The prediction capacity of LRRC39 is validated by PCR. (A) qRT-PCR showing the expression of LRRC39 in RB is remarkedly higher than that in invasive RB. *P* value is determined by student’s *t* test. The relative expression of LRRC39 was presented as fold change (log2 2^−ΔΔCT^). (B) Kaplan-Meier curve showing the survival outcome of RB patients with different LRRC39 expression. Log-rank test, *p* = 0.02.

Considering that LRRC39 had the best discrimination accuracy, we we performed qRT-PCR for it to further confirm LRRC39 has clinical utility. As expected, we found LRRC39 in invasive RB were significantly lower than that in RB (Fig. 7A). More importantly, patients with higher expression of LRRC39 had more favorable outcomes as compared with those with low expression of LRRC39 (Fig. 7B). These results suggested LRRC39 would be a promising stratification biomarker for RB.

## Discussion

Although RB1 loss is regarded as the susceptible malignant factor, it only has an effect on developing from retinocytoma to RB. For these RB without RB1 loss, we still know little about the biological process of RB tumorigenesis (Dimaras et al., 2012). A few studies recently unveiled the heterogeneity of RBs and subtypes of RB was uncovered (Cobrinik, 2015). However, there is no consensus on the subtype of RBs and whether some subtypes of RB are prone to be progressive is still unclear. Here, we utilized two public datasets to generate the RB progression signatures that indicate the risk of RB progression from RB to invasive RB. Additionally, we clustered RBs into four subtypes on their genes expression and uncovered one of the subtypes is less progressive than the other three subtypes by other two public dataset. Compared RB subtype genes with the RB progression signatures, we shortlisted five RB subtype progression signatures (CLUL1, CNGB1, ROM1, LRRC39 and RDH12). These five RB subtype gene signatures were also demonstrated the predictive capacity for RB progression. Of the five RB subtype signatures, LRRC39 revealed the best predictive accuracy and was validated in our clinical cohort. Studying the RB progressive signatures therefore may improve our understanding of RB heterogeneity and progression.

In a retrospective histopathological review, Eagel et al. investigated on 297 primary enucleated eyes, and found the inverse relation between photoreceptor-related differentiation and age at enucleation (Eagle et al., 2009). In this pathological review, differentiated and undifferentiated cells can co-exist within the same tumor, which suggests there is a large intratumor heterogeneity in RB. Undifferentiated tumors are related with high expression of mitosis related genes (Eagle et al., 2009). This suggests that higher expression of the cell cycle related signature could have been a result of lower proportions of more differentiated and higher proportions of less differentiated cells. It has also been advised that differentiated areas in RB are likely benign precursor lesions of the undifferentiated areas (Dimaras et al., 2008). Dimaras et al. disclosed that differentiated eosinophilic areas are associated with bi-allelic inactivation of RB1, while adjacent basophilic undifferentiated areas require additional genetic lesions to accommodate rush tumor progression. When additional genetic lesions and the dysfunction of cell cycle regulation occur after late RB1 inactivation, the highly proliferative cells are likely to expand rapidly, ultimately leading to tumors with relatively few differentiated cells.Therefore, restoration of normal cell cycle regulation and induction of differentiation in precursor lesions would potentially be the targets of RB treatments.

Our results of RB progression signatures indicate the dysfunction of cell cycle and loss of retinal related cell differentiation play critical roles in RB progression, which is consistent with previous studies. Kooi et al. also revealed rare RB driven by MYCN amplification rather than RB1 loss (Kooi et al., 2015; Rushlow et al., 2013). Another study also demonstrated MYCN amplified RB tumors that having small proportion of all RBs have distinct histology, early onsets and probably more aggressive and poor outcomes. Although dysregulation of cell cycle presented in a limited subpopulation of RB, our results showed cell cycle broadly involved in the tumorigenesis and progression of RB. Our results also further confirmed loss of control of retina cells differentiation is the key feature of RB progression and invasion. The enriched pathways of distinct genes between normal retina, RB and invasive RB highlighted the differentiation induction of undifferentiated retina cells would be the potential therapeutic target in the early stage of RB.

Stratification of heterogonous tumors enables clinicians to predict disease progression risk and precision medicine to be applied in individual treatment. Two main subtypes (photoreceptor and undifferentiated subtypes) were identified in previous studies regarding RB transcriptomes. Subtype of RB containing high expression of photoreceptor gene signatures is regarded with well differentiation of retina cells.

RNA signatures have been determined in several distinct RB cohorts (Chakraborty et al., 2007; Ganguly & Shields, 2010; Kapatai et al., 2013; McEvoy et al., 2011). Kapatai et al. (2013) performed unsupervised hierarchical clustering on genome-wide expression estimates of 23 RB samples. Similar to Ward’s clusters RB subtype 1, 2 and 3 (Loss of photoreceptorness and gain of genomic alterations in RB reveal tumor progression), three RB groups including a small (*n* = 2) retina-like group were identified by Kaptai et al. Explanation for the identified differences in RB genes expression was mainly focused on the cell of origin. Kapatai’s group 1 tumors were described to originate from retinal progenitor cells (RPCs) while group 2 tumors from a cone photoreceptor lineage. However, Xu et al. (2014) found that only cone photoreceptor precursors become proliferative subsequent to RB1 loss and are likely the only cells that can transform into RB, suggesting the heterogeneity of RB and fast-cycling cells are common in subpopulation of RBs. Furthermore, they also suggested that RB1-deficient cone precursors form differentiated RBs that subsequently dedifferentiate and acquire non-cone characteristics, which highlights the regulatory abnormality of cell cycle and differentiation probably spans across all subtypes of RBs. Although a few subtype stratifications of RBs have been explored in some independent cohorts, the relationship of RB subtypes and RB progression hasn’t been fully investigated, where whether undifferentiated RB subtypes are prone to be more progressive and additional adjuvant therapeutics are required.

In order to explore whether one specific RB subtype is more invasive than other subtypes, we integrated two public datasets that involves more than 100 RBs in total and correlated RB progression gene signatures with RB subtypes. We identified the distinct progression gene signatures patterns in four subtypes of RBs and found subtype4 had less progressive gene signatures of all subtypes of RB. This result indicates favorable outcomes of patients would likely be associated with subtype4 RBs and observations rather than adjuvant therapeutics could be applied for this particular group in future clinical practice. In addition, we demonstrate a panel of five subtype featured gene signatures (CLUL1, CNGB1, ROM1, LRRC39 and RDH12) have predictive capacity of RB progression with outstanding accuracy. Our qRT-PCR results confirmed LRRC39 is potentially the progressive biomarker for RB with the highest accuracy. We also noticed there are some limitations in our study. Our study is a bioinformatic analysis and it still needs to be validated in a large cohort. Lack of the clinical information such as the survival information, we rarely corelated our results with patients’ survivals. Further investigation we are going to do is to collect the follow-up information of RB patients and test whether LRRC39 is a prognostic biomarker for RB. Although these five genes need to be further tested in a large cohort, they shed a light on the early detection of invasive RB and risk stratification of RBs. In addition, we observe there is a large difference of subtype specific genes between our study and previous studies and only 5% of genes are overlapped (Fig. S7; Table S3). In contrast to the two subtype stratification studies revealed a small subpopulation of RB is enriched with lymphocyte markers, we did not identify immune cell infiltration related markers in our DE gene sets. That is mainly caused by the different subtype clustering algorithm we used. Another reason probably is due to the bias of the included cohort population, where we involved 131 RB patients into our analyses. Mechanically, immune cell infiltration and microenvironment interaction with RB is likely observed in all types of RB, where immune cells markers present in orthotopic transplantation models of RB in mice and immunostimulatory factors such as IL-12p70, TNF-α, IL-6, IL-1β and IL-8 are secreted by RB cells to accommodate immune cells in the microenvironment (Lee et al., 2013; Ma et al., 2014).

Although the histopathology-based staging and stratification is well established and recognized globally, molecular biomarkers have revealed it unique value as a companion test for cancer diagnosis, stratification, therapeutic regimen selection and prognosis in the era of precision medicine (Bouras et al., 2019; Dudley et al., 2016; Ma et al., 2018; Malapelle et al., 2017). Our proof-of-concept study showed the expression of LRRC39 is strongly correlated with RB cell differentiation and favorable outcomes of patients with RB. Our independent cohort also validated the clinical utility of LRRC39 as a predictive biomarker. In future, LRRC39 test would potentially be the risk stratification and therapeutic biomarker. Ideally, it could allow physicians to decide whether the additional adjuvant therapy should be applied according the results of LRRC39 test. To achieve this aim, a large biomarker driven cohort that investigating the clinical utility of these biomarkers should be conducted locally or collaboratively.

Our studies also have a few limitations due to the scale of our cohort. First, a large validation cohort is needed to further examine the clinical utility of our subtype stratification and subtype progression gene signatures for RBs. Second, due to lack of the outcomes information of each sample from GEO datasets, we failed to test the prognostic capacity of these RB progression signature (Chen et al., 2018), which requires another large cohort involving more comprehensive treatment and outcomes information to validate the progression signatures in RB prior to the application in clinical practice. Last, we also haven’t compared our results with the results from single cell sequencing of retina but a further correlation analyses will be conducted to unveil whether the progression and subtype of RB is the proxy of retina development (Macosko et al., 2015).

## Conclusions

In summary, the results of this study discover the critical gene signatures during progression of RB. Impairment of cell cycle regulation leads to the RB progression and invasion. Additionally, a comprehensive dataset is employed for RB subtype stratification, where four subtypes of RB is identified. Subtype featured gene signatures show loss of control of cell cycle and cell differentiation is main difference between RB subtypes. One RB subtype with low expression of cell cycle genes and high expression of visual perception demonstrates progressive or invasive feature of all four subtypes of RB. Five-gene signature reveals the outstanding predictive power of high-risk of RBs in the proof-of-concept study. Of them, LRRC39 would be the most promising predictive biomarker for RB progression.

## Supplemental Information

10.7717/peerj.8873/supp-1 Supplemental Information 1 A heatmap of differentially expressed genes in GSE118011.

10.7717/peerj.8873/supp-2 Supplemental Information 2 A combination of two independent public datasets for RB subtype identification. A, boxplots of the gene expression of each included samples before (left) and after (right) batch correction. Green, GSE29683; blue, GSE59983. b, PCA plots showing the variance of RB labeled by batches (left) or subtypes (right) using top 2,000 variable genes. Each dot represents each RB samples.

10.7717/peerj.8873/supp-3 Supplemental Information 3 Bubble plots of the enriched GO (left) and KEGG (right) pathways of subtype specific genes (subtype1, subtype2 and subtype3).

10.7717/peerj.8873/supp-4 Supplemental Information 4 The heatmap showing the subtype genes can identify invasive RB.

10.7717/peerj.8873/supp-5 Supplemental Information 5 Bar plots of enriched pathways across input gene lists, colored by *P*-values. Upper, first eight genes clustered in Fig. 4; lower, last 13 genes clustered in Fig. 4.

10.7717/peerj.8873/supp-6 Supplemental Information 6 Violin plots of five RB progressive subtype signatures in dataset GSE97508 (upper) and GSE110811 (bottom).

10.7717/peerj.8873/supp-7 Supplemental Information 7 Venn diagram showing the overlap gene of RB subtype genes in our study and previous study.

10.7717/peerj.8873/supp-8 Supplemental Information 8 Retinoblastoma progression signatures.

10.7717/peerj.8873/supp-9 Supplemental Information 9 RB subtype specific genes.

10.7717/peerj.8873/supp-10 Supplemental Information 10 The statistical results of a post hoc test (Tukey HSD test) for 5 gene signatures.

10.7717/peerj.8873/supp-11 Supplemental Information 11 The comparison of subtype specific genes between our study and previous study.

## Additional Information and Declarations

### Data Availability

The following information was supplied regarding data availability: Data is available at NCBI GEO: GSE97508, GSE110811, GSE29683 and GSE59983.
