# Supplementary figures and images for: Bioinformatics analyses of retinoblastoma reveal the retinoblastoma progression subtypes
Source: PeerJ. 2020 May 21;8:e8873. doi: 10.7717/peerj.8873 (PMC7246025; doi:10.7717/peerj.8873)

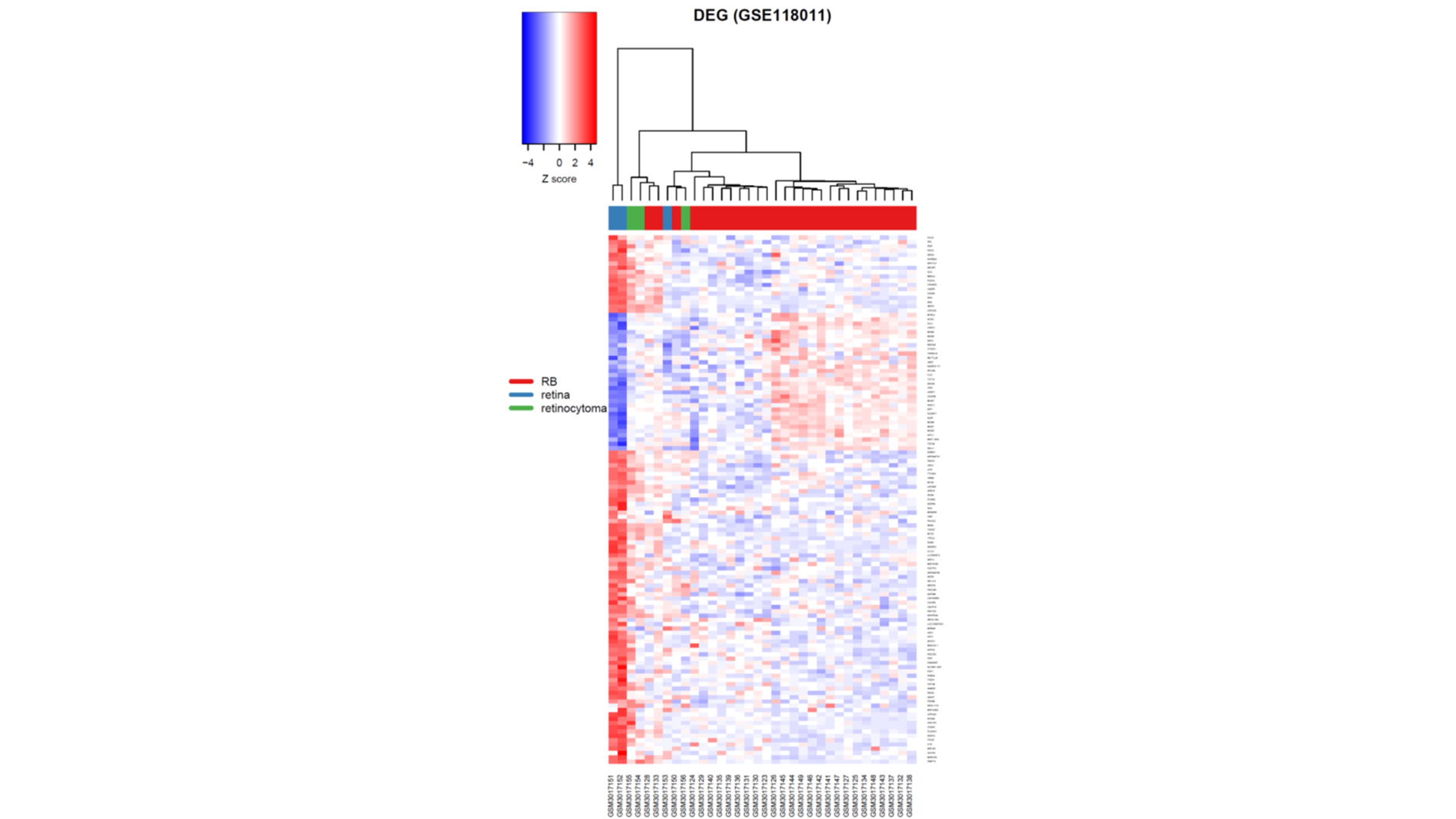

Supplement: Supplemental Information 1 [file peerj-08-8873-s001.png]

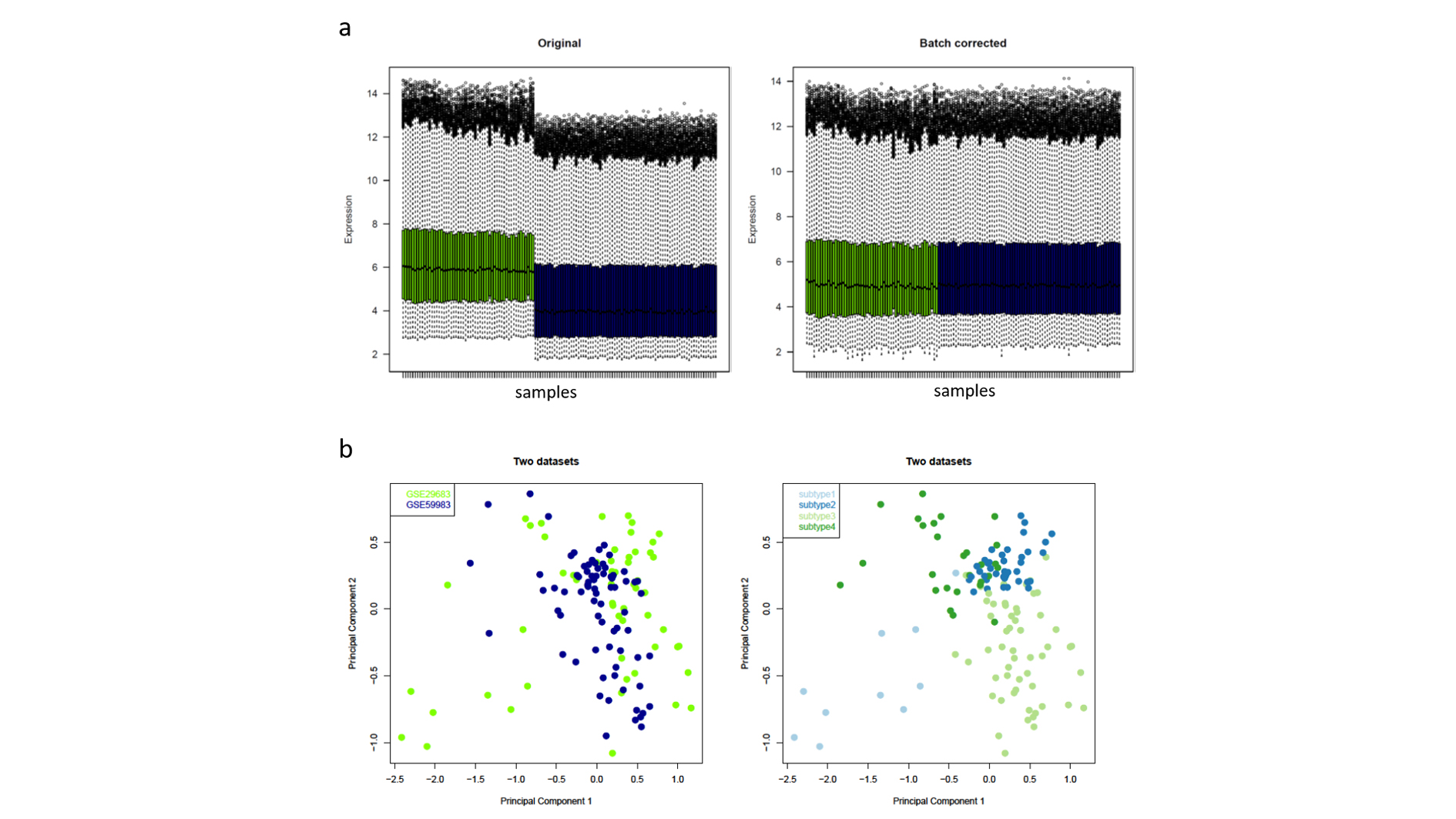

Supplement: Supplemental Information 2 — A, boxplots of the gene expression of each included samples before (left) and after (right) batch correction. Green, GSE29683; blue, GSE59983. b, PCA plots showing the variance of RB labeled by batches (left) or subtypes (right) using top 2,000 variable genes. Each dot represents each RB samples. [file peerj-08-8873-s002.png]

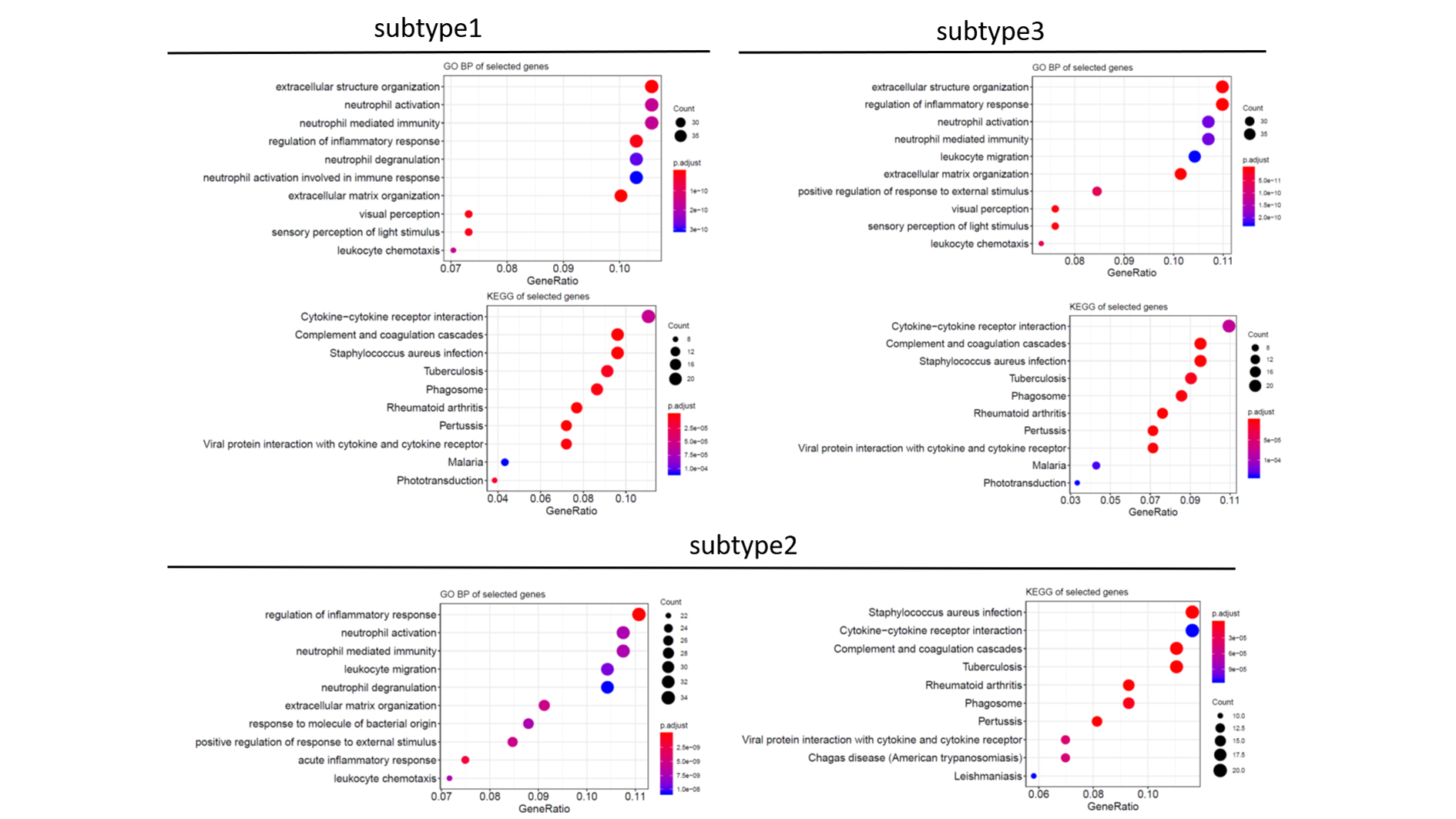

Supplement: Supplemental Information 3 [file peerj-08-8873-s003.png]

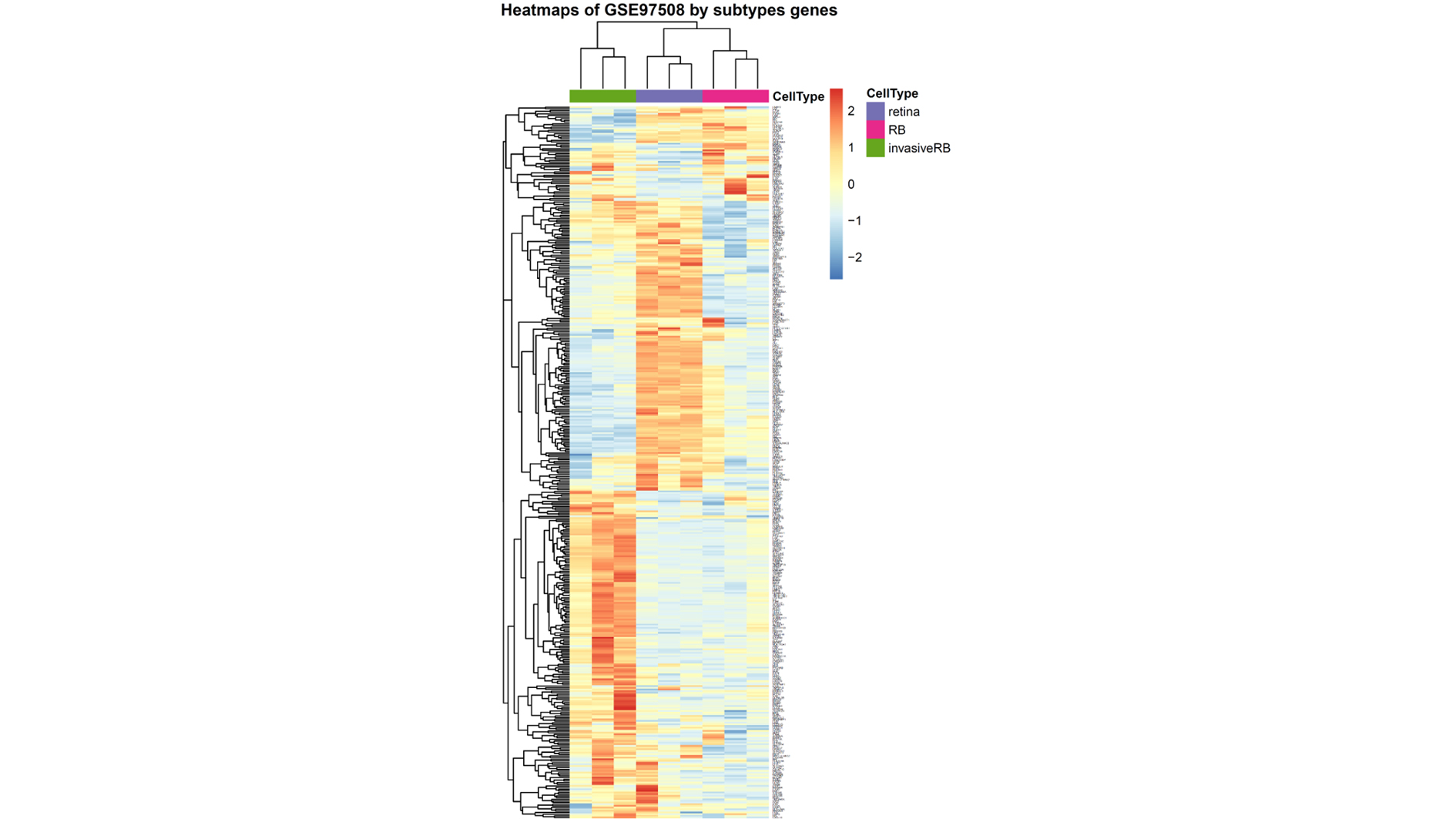

Supplement: Supplemental Information 4 [file peerj-08-8873-s004.png]

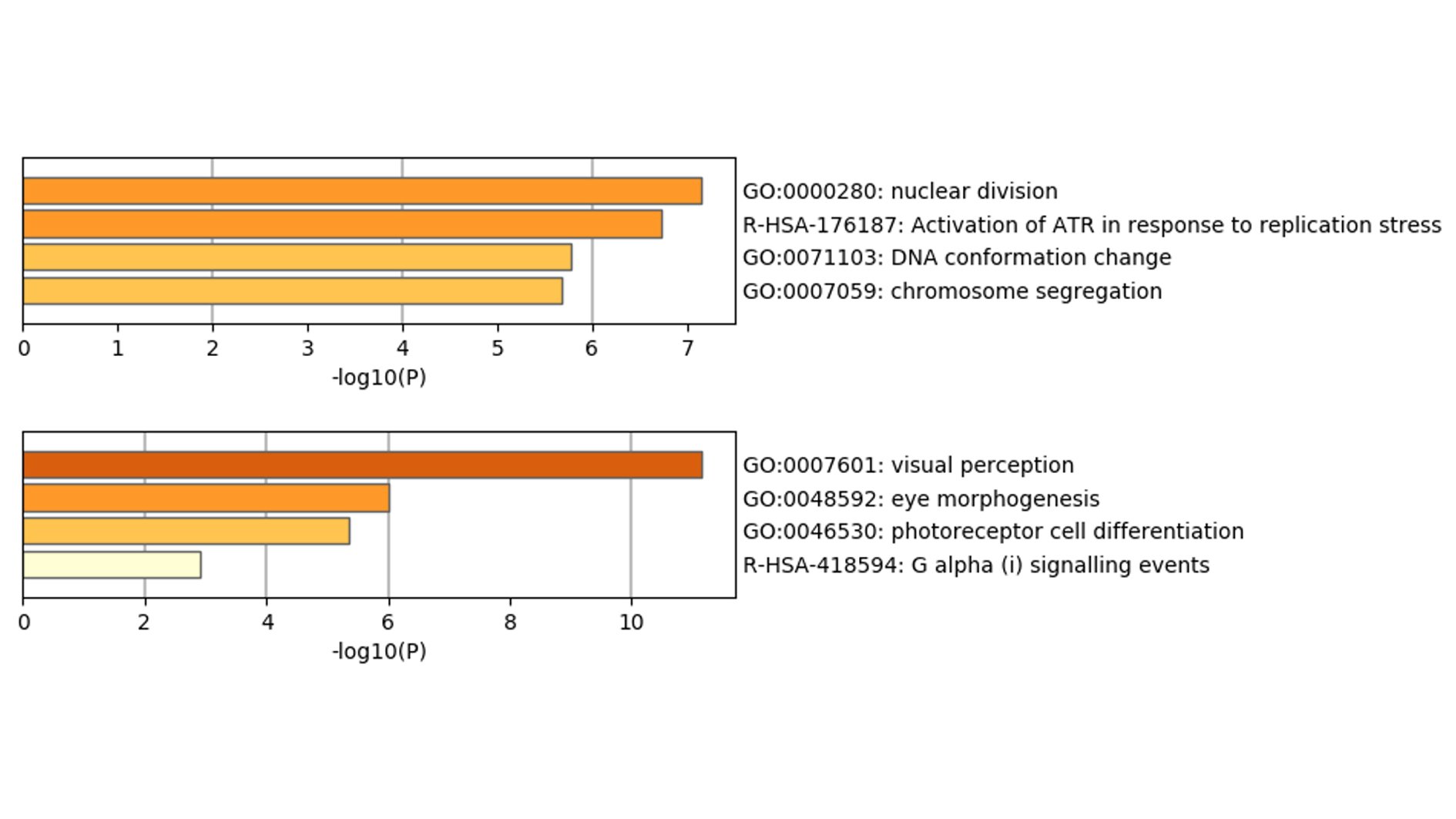

Supplement: Supplemental Information 5 [file peerj-08-8873-s005.png]

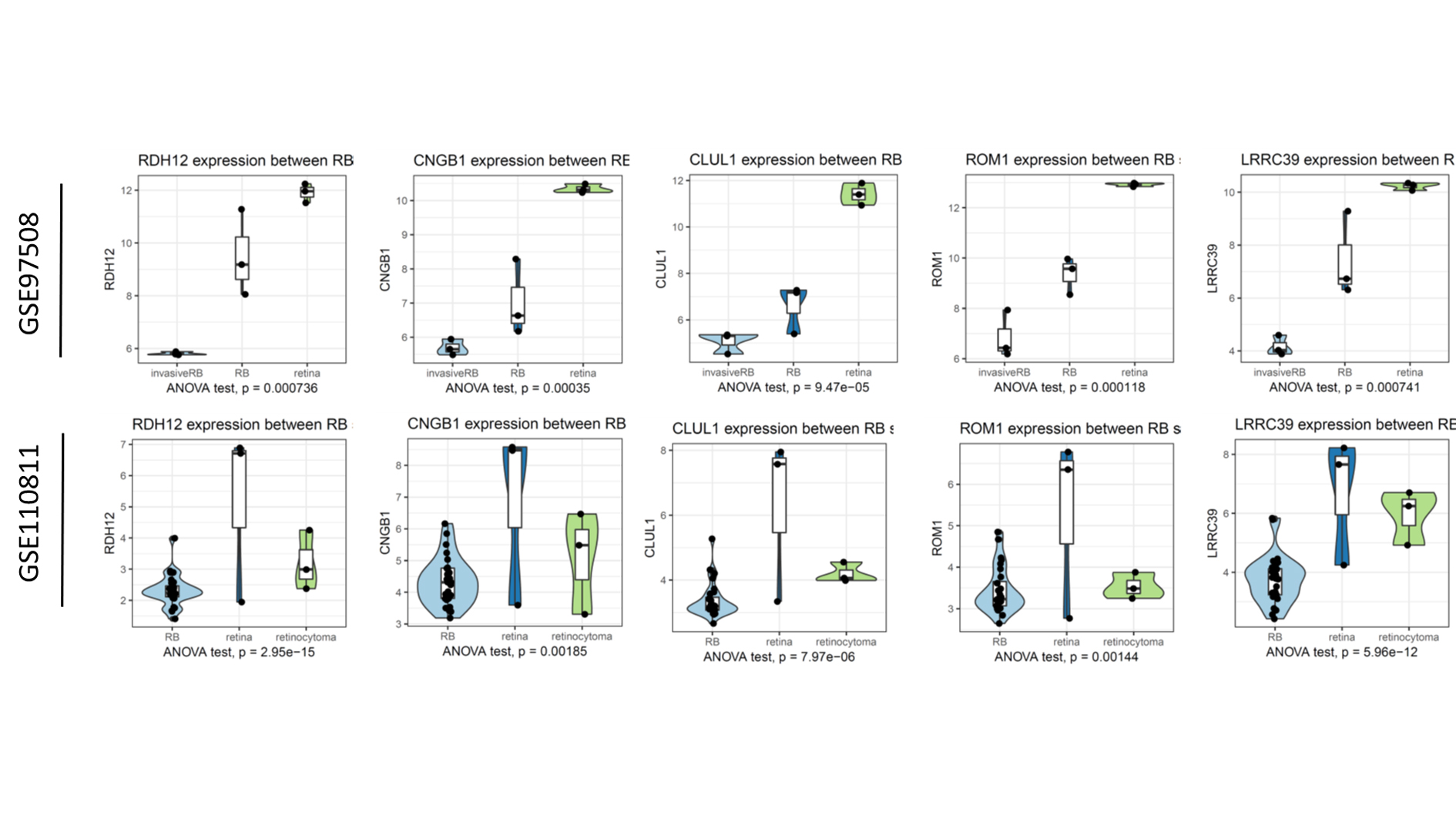

Supplement: Supplemental Information 6 [file peerj-08-8873-s006.png]

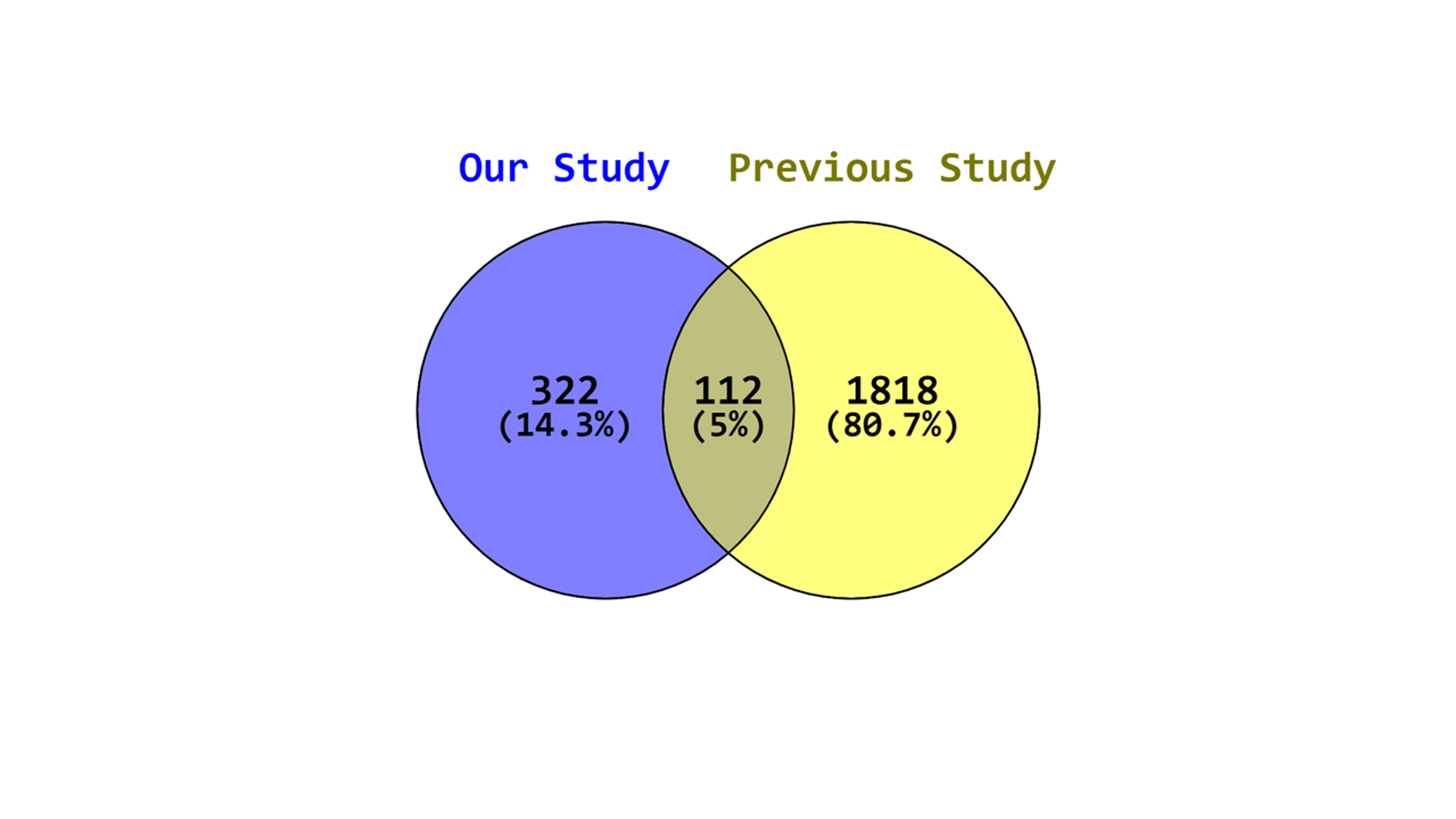

Supplement: Supplemental Information 7 [file peerj-08-8873-s007.png]
